# Phyllodes Tumor, A Cytomorphologic Study of 17 Cases with Histologic Correlation

**DOI:** 10.30699/ijp.2024.2033852.3322

**Published:** 2025-01-10

**Authors:** Savita Agarwal, Pinki Pandey, Megha Sawhney, Alka Yadav, Sunita Kumari Meena

**Affiliations:** 1 *Department of Pathology, Uttar Pradesh University of Medical Sciences, Saifai, Etawah, India *; 2 *Department of Pathology, Post Graduate Institute of Child Health Noida, Uttar Pradesh*

**Keywords:** Cytohistologic correlation, fibroepithelial tumor, FNAC, Phyllodes

## Abstract

**Background & Objective:**

Phyllodes tumor (PT) is a rare fibroepithelial tumor of the breast exhibiting varied clinicopathologic behavior, ranging from benign to borderline to frankly malignant, based on the presence of infiltrative margins, stromal overgrowth, stromal atypia, cellularity, and mitotic activity. In this study, a detailed cytomorphological study of cases of PT with the clinical and histological correlation was performed.

**Methods:**

A cytomorphological study of 17 cases of histologically proven PT diagnosed between Jan 2014 and July 2021 was done retrospectively. Relevant data including age at the time of diagnosis, the duration of illness, presenting symptoms, personal and family history, tumor size, tumor localization, and surgical procedure were obtained. A detailed cytomorphological assessment of stromal and epithelial components was performed, and further histological correlation was obtained for each case.

**Results:**

Age of the patients ranged from 25 to 65 years old. The chief complaint was a palpable breast mass in all patients. The mean size of the lump was 11.86 cm. A complete cytohistological concordance was achieved among malignant cases. Stromal metaplasia was observed in only one case of benign phyllodes tumor, which was chondroid differentiation, and malignant heterologous component as fibrosarcomatous differentiation in one of the malignant PTs. Each of the benign and malignant phyllode tumors ductal carcinoma in situ (DCIS) of its epithelial component was seen in one case.

**Conclusion:**

Phyllodes should be considered in differential diagnosing of any rapidly growing breast lump. Breast imaging has limited role in diagnosis of phyllode tumors. FNAC or trucut biopsy is mandatory in preoperative diagnosis. An extended follow-up is needed in all cases.

## Introduction

Breast pathologies are among the most common lesions subjected to fine needle aspiration (FNA) for initial diagnosis, with carcinoma and fibroepithelial neoplasms comprising the majority. The latter includes fibroadenoma (FA) and phyllodes tumor (PT). Phyllodes tumors are rare fibroepithelial lesions, accounting for 0.3% to 1% of all breast tumors (1). While fibroadenomas are considered benign, the behavior of phyllodes tumors is highly unpredictable. Some cases behave as benign tumors and are amenable to simple excision, whereas others can metastasize or recur, requiring more aggressive treatment (2). Therefore, accurate preoperative diagnosis is essential to guide appropriate management. However, the cytologic features of fibroadenoma and benign phyllodes tumors show significant overlap (3).

We retrospectively analyzed 17 cases of phyllodes tumors for which both cytology smears and follow-up histopathology sections were available. The sensitivity and specificity of both methods in diagnosing phyllodes tumor were evaluated.

## Material and Methods

The present retrospective study was conducted on a total of 17 cases of histologically proven phyllodes tumors diagnosed between Jan 2018 and July 2023 in which prior FNA was performed and smears were available for review. The cytology smears were reevaluated without the knowledge of the initial cytologic diagnosis, and an attempt was made to sub-classify the cases into benign, borderline, and malignant phyllodes. A detailed study of cases was undertaken for epithelial and stromal components, where the proportion, cellularity, and atypia of both the components were evaluated in additionto stromal-epithelial ratio, percentage of single scattered spindle cells in the background, and mitosis. For the number of fragments, a cut-off of less than and more than 05 fragments in the smear was used to indicate few fragments and many fragments, respectively for both the epithelial and stromal components as adopted by Krishnamurthy* et al. *(4). Stromal cellularity and atypia were assessed on the scale of 1+,2+, 3+ to represent mild, moderate and marked respectively. Mitosis was assessed as 1+ (<2/10hpf), 2+ (2-4/10 hpf), and 3+ (>4/10 hpf), similar to the criteria used by Bhattarai S* et al. *(5). Epithelial fragment architecture and stromal to epithelial ratio were also assessed. The stromal-to-epithelial ratio was evaluated as stromal predominant, epithelial predominant, or both components equally prevalent. Single scattered stromal cells were categorized as > 10%, 10-30%, and >30% as by Krishnamurthy* et al. *(4). To count the number of epithelial and stromal fragments, 10 medium power (10X) fields were observed, and an average was taken. Cytology slides available for review were Giemsa, hematoxylin, and eosin, and Papanicolaou stained, and histology slides were hematoxylin and eosin stained. The numbers of epithelial and stromal fragments were counted on 10 medium power fields, and an average of ten fields was taken as by Maritz* et al. *(6). The data was compiled in an Excel sheet and using SPSS software (SPSS Inc., Chicago, Ill., USA), the Fischer exact test was applied to calculate the P-value amongst various cytological parameters. A P-value of less than 0.05 was considered statistically significant.

### Observation

The present study consisted of 17 cases of histologically proven phyllodes tumors with an age range of 22-63 years and a mean age being 44.06 years. The size of the lump ranged from 3.5 cm to 5.8 cm with the mean size being 4.2 cm. Table 1 shows the clinical characteristics of all the patients with phyllodes tumors.

Table 2 shows the correlation between cytological and histological diagnoses of all cases.02 cases were reported as fibroadenoma on cytology and turned out to be benign phyllodes on histology. The correlation was not statistically significant (*P*=0.48). Considering histopathology as the gold standard, the sensitivity of cytology in diagnosing malignant phyllodes tumors was 100%. The sensitivity and specificity of cytology in diagnosing benign and borderline PT was 87.5% and 100%, respectively.

## Discussion

Phyllodes tumor was first described by Johannes Muller in 1838 in Germany (7). Fibroadenoma (FA) and benign phyllodes tumor (BPT) share similar morphologic features, as both exhibit dimorphic patterns comprising stromal and epithelial components. Consequently, distinguishing between the two in cytology can be challenging (8). FNA is a commonly used first-line preoperative test in the investigation of palpable breast masses. For diagnosis of breast carcinoma, its sensitivity is reported to be 100% when combined with the clinical and mammography results (9). But its sensitivity ranges from 32% to 77% for diagnosis of phyllodes (10). Malignant phyllodes can be diagnosed on FNAC with the prominent stromal component, marked atypia, pleomorphism, and high mitotic count. The main problem arises in diagnosis of benign and borderline phyllodes cases (11). Phyllodes were considered to be benign, until in 1943, Cooper and Ackerman reported its malignant counterpart (12). In 1981, World Health Organization (WHO) classified these tumors into benign, borderline, and malignant as given in Table 4.

On histopathology, features mentioned in WHO criteria can be evaluated but on FNAC, features like tumor margins cannot be assessed. Also, there might be focal atypia and focal mitotic focus that may be missed on FNAC due to limited sampling. In our study, 88.2% of phyllodes cases showed stromal fragments >5, differentiating fibroadenoma from phyllodes. However, this was not a significant criterion to distinguish between benign, borderline, and malignant cases (p=0.47). Similar results were obtained in studies by Scolyer* et al., *El Hag* et al., *Maritz* et al., *Veneti* et al., *Jayaram* et al., *and Bandyopadhyay* et al. *(6,11,13,14,15,16). Low stromal cellularity was seen in benign PT and all malignant PTs showed high stromal cellularity in our study. In numerous previously reported s studies, presence of hypercellular stroma was one of the most distinguishing features between fibroadenoma and phyllodes. In a study by Krishnamurthy* et al. *and Maritz* et al. *approximately 30% of cases of fibroadenomas showed hypercellularity and so they concluded that this feature cannot be used unequivocally for diagnosing phyllodes (4,6).

In the present study, marked stromal atypia was seen in borderline and malignant PTs, while no atypia was seen in the benign PTs category. No significant association was seen between the degree of atypia and categories of phyllodes tumors. In a study by Maritz* et al., *there was a significant association between stromal atypia in fibroadenomas and phyllodes (6). Stromal atypia was found to be an important feature in studies by Bhattarai* et al., *Krishnamurthy* et al., *El Hag* et al., *and Rao* et al. *(4,5,11,18).

Shimizu K emphasized the patterns of the epithelial component in differentiating between BPT and fibroadenoma where tubular, monolayered, and blunt branching small to medium-sized fragments favored fibroadenoma and folded and wavy large-sized (> 1mm in diameter) epithelial fragments were seen in BPT (19). In our study, phyllodes tumors mainly showed a wavy pattern with 02 cases of BPT showing monolayered sheets and hence were misdiagnosed as fibroadenoma on FNAC. The behavior of BPT is also unpredictable as some cases may show a recurrence which is largely attributable to incomplete surgical excision (20,21). Rarely it undergoes malignant transformation and hence correct preoperative diagnosis is essential for clinical management (20). In a study by Ashfaq* et al., *they found that stromal cells in PTs are larger and wavy while in fibroadenomas they are small and round to oval (22). However, they found no significant association between these features and PTs. Another study by Deen* et al. *also didn’t find any significant difference between in type of stromal fragments and FAs, variants of FAs, and BPTs (23).

In our study, phyllodes tumors showed a lesser number of epithelial fragments, and the stromal-to-epithelial ratio was higher in malignant PTs than in BPTs. However, this difference was not significant (*P*=0.21). In a study by Bandyopadhyay* et al., *the S:E ratio was found to be an important factor in distinguishing FAs from PTs, while in a study by Bhattarai* et al., *it was considered an important feature in distinguishing three grades of PTs (5,16).

In our study, the presence of singly scattered stromal cells showed a significant association in differentiating three grades of phyllodes. The presence of >30% single scattered cells was seen in 84% of malignant PT cases while 02 cases of BPT showed <10% scattered spindle cells. According to Krishnamurthy* et al., *<10% of spindle cells favor the diagnosis of FA, and >30 % favor PTs, while 10-30% indicate the intermediate zone (4). However, El Hag differed in this cut-off and concluded in their study that, a background of 10% spindled cells rather than 30% would increase sensitivity in diagnosing phyllodes tumors and will reduce the possibility of misdiagnosing PT as FA (11). Our study and study by Maritz* et al. *(6) favor that cut-off given by Krishnamurthy* et al. *are better discriminator of these tumors. 

Two cases in our study were wrongly diagnosed as fibroadenoma instead of BPT on cytology. This may be because of sampling error as the diagnostic area was focally present in the tumor and may be missed on FNAC. Therefore, a thorough sampling of all breast lesions should be done to improve diagnostic accuracy. Clinical and radiological features alone cannot reliably distinguish fibroadenoma and phyllodes tumor and FNA is frequently used as a first-line investigation for breast lumps (6,24). 

It can be difficult to distinguish between a PT and a fibroadenoma using FNA due to overlapping features. Lack of familiarity with cytological features, disease rarity, morphological heterogeneity, and inadequate sampling can lead to poor FNAC outcomes. While individual cytological parameters may not be decisive, when considered together, they can effectively differentiate between the two groups (25).

Kumar PV*et al* in their study reported that the presence of stromal hypercellularity, bonsai-like epithelial clusters, amorphous pinkish material at the border of stromal fragments, intranuclear inclusions, and popcorn-like nuclei in stromal cells may aid in diagnosing phyllodes tumors (26).

In FNA smears features favoring a malignant PT include high stromal cellularity, prominent stromal nuclear atypia, presence of mitotic figures, scattered single atypical cells, multinucleated tumor giant cells, and heterologous differentiation of sarcomatous stroma with features of osteosarcoma, liposarcoma, chondrosarcoma, or rhabdomyosarcoma (27).

Kuppusamy DA reported a case of a 58-year-old woman who presented with a large breast mass and on FNAC showed features consistent with malignant PT, with prominent areas of heterologous liposarcomatous differentiation. The cytological findings were also confirmed by histology. Authors concluded that malignant PT and its different tissue components can be accurately diagnosed through FNA cytology when performed optimally. This can be crucial for preoperatively assessing patients suspected of having malignancies to plan surgical procedures accordingly (28). Li JJ* et al. *presented a case of 54-year-old woman with recurrent malignant phyllodes tumor that metastasized to left pleura leading to massive unilateral malignant pleural effusion (29). PTs are rare and difficult to diagnose preoperatively. Grading them pathologically is important for predicting recurrence and survival rates. Benign and borderline PTs have a less aggressive course than malignant PTs. Excision with negative margins is the recommended treatment. The role of adjuvant radiation therapy in borderline and malignant PTs needs further investigation (30).

The main limitation of our study is the low number of cases. This is because of the rarity of PTs, as they constitute only 1 % of breast tumors.^1^ Another limitation would be the lack of definite criteria to describe atypia, counting stromal and epithelial fragments and counting single scattered stromal cells amongst various studies done so far. Due to the limited amount of material in core biopsies and FNACs, there are difficulties in obtaining a correct diagnosis. To reduce diagnostic inaccuracy, vacuum-assisted breast biopsy (VABB) was introduced in 1995. This helps in more accurate diagnosis and complete removal of the lesions with the help of real-time USG (31).

**Table 1:** Clinical characteristics of the patients with phyllodes tumor.

Total No. of cases	17
Age range (years)	22- 63
Mean age	44.06
Gender	Females (n=17)
Laterality	Right= 06, Left=11
Duration of a lump in months (mean)	4.3
Size range (cm)	3.5- 5.8
Surgical procedure	Wide excision (n=10) Simple mastectomy (n=01) MRM (n=06)
Outcome	Local recurrence (n=0), Metastasis (n=1)

**Table 2:** Cytohistological correlation.

Cytopathology (n= 17)	Histopathology (n= 17)		
	Benign PT (08)	Borderline PT (03)	Malignant PT (06)
Benign PT = (07)	06	01	-
Fibroadenoma = (02)	02	-	-
Borderline PT = (02)	-	02	-
Malignant PT= (04)	-	-	04
Malignant PT/ Metaplastic carcinoma = (02)	-	-	02

**Fig. 1:**
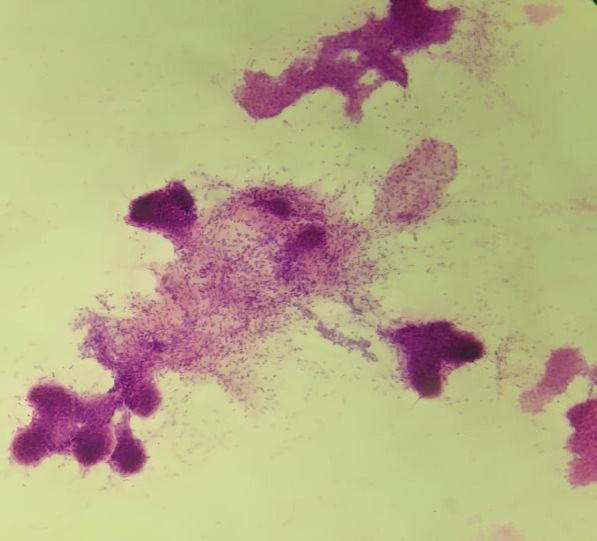
FNA smears of benign phyllodes tumour showing prominent epithelial and fewer stromal fragments. (H/E 40X).

**Fig 2:**
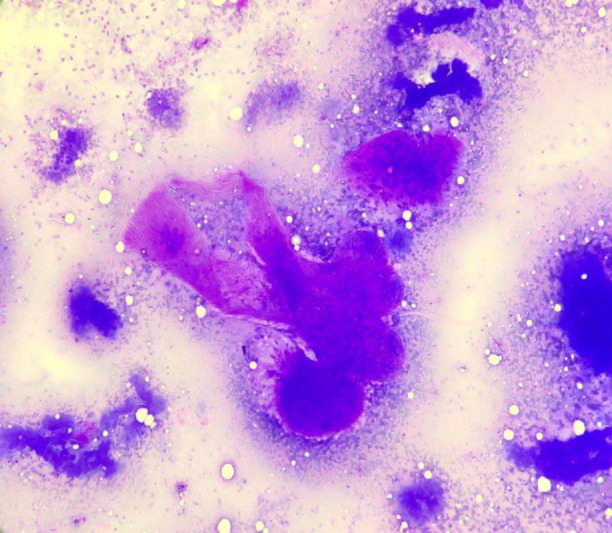
FNA smears of benign phyllodes tumour showing more than five epithelial fragments and low stromal to epithelial ratio (MGG 40X).

**Fig 3:**
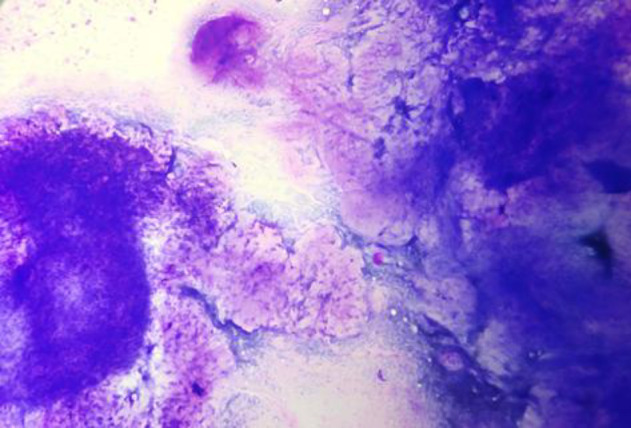
FNA smears of benign phyllodes tumour showing marked myxoid degeneration in stromal fragments. (MGG 40X).

**Fig 4:**
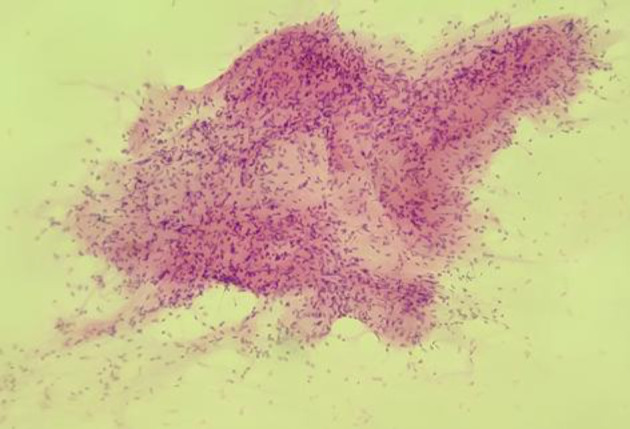
FNA smear with moderate stromal cellularity and pleomorphic stromal cells. (H/E200X)

**Fig 5:**
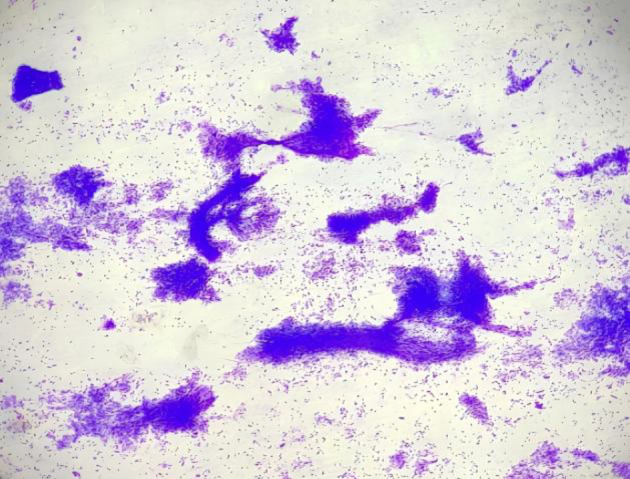
FNA smear of malignant Phyllodes tumor showing high cellularity with predominantly stromal clusters. (MGG100X).

**Fig 6:**
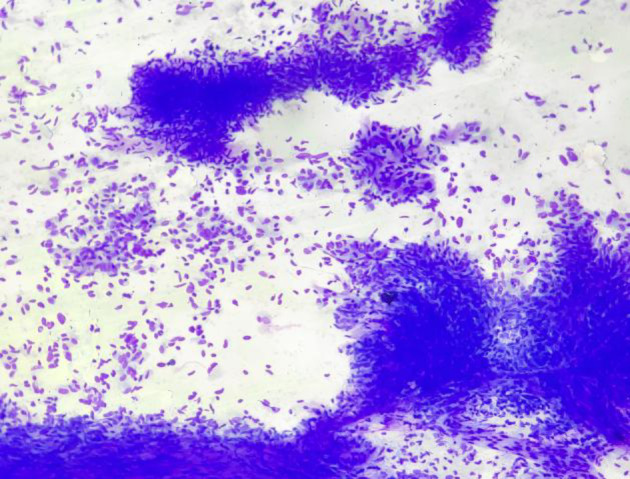
FNA smear of malignant Phyllodes tumor showing numerous singly scattered stromal cells. (MGG 200X)

**Fig 7:**
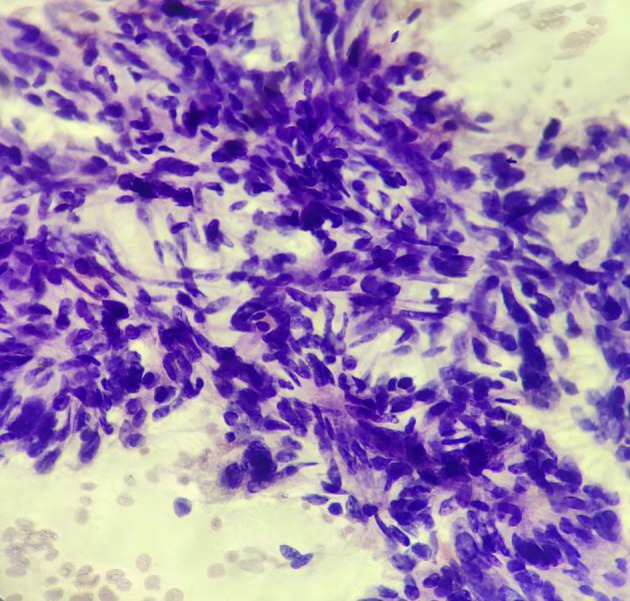
FNA smear of malignant Phyllodes tumor showing highly cellular cluster of malignant stromal cells with nuclear hyperchromasia and pleomorphism. (PAP400X).

**Fig 8:**
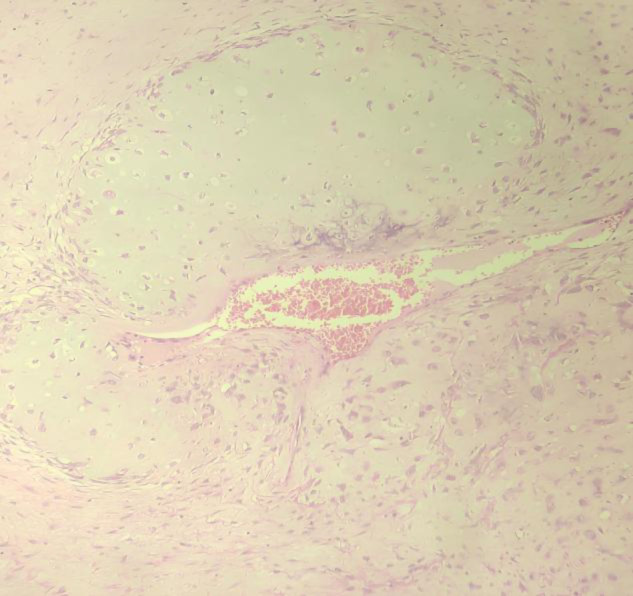
Section from benign Phyllodes tumor showing foci of chondroid differentiation. (H/E 100X)

**Fig 9:**
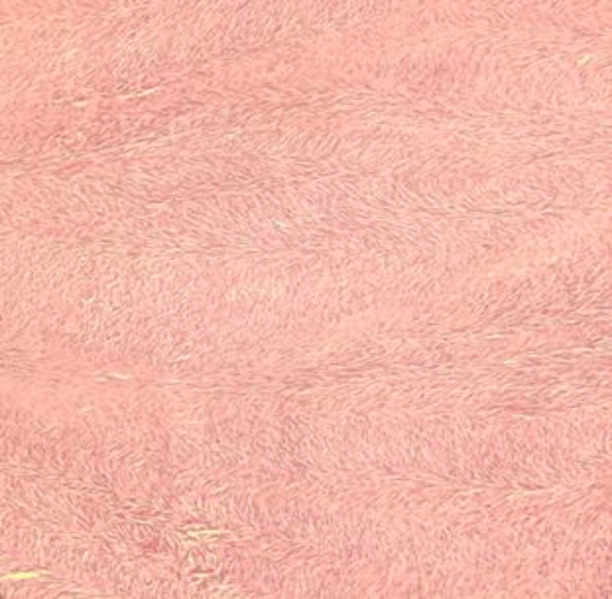
Section from Malignant Phyllodes tumor with fibrosarcomatous area. (H/E 100X)

Table 3 shows cytomorphological characteristics observed in the present study (Figure 1-7). There was no significant association between the number of stromal fragments, atypia, or number of epithelial fragments (*P*=0.47, *P*=0.08, *P*= 0.21 respectively) with benign, borderline, and malignant phyllodes category. Only a statistically significant association was found between singly scattered stromal cells and three categories of phyllodes tumors (*P*=0.012). On histopathology, stromal metaplasia was observed in only one case of benign phyllodes tumor, which was chondroid differentiation (Figure 8) and malignant heterologous component as fibrosarcomatous differentiation (Figure 9) in one of the malignant PTs. In one case each of the benign and malignant phyllodes tumors ductal carcinoma in situ (DCIS) of its epithelial component was seen.

**Table 3:** Details of cytomorphological analysis with the case-wise distribution.

Histopathology (n= 17)		Cytopathology (n= 17)			Cytologic features																			
					Stromal Fragments																			
					Number					Stromal cellularity						Atypia								
					<5 (Few)		>5 (Many)			+		++		+++		-		+			++		+++	
Benign PT (08)		Benign PT = (06)			-		06			01		05		-		01		05			-		-	
		Fibroadenoma = (02)			-		02			01		01		-		01		-			-		-	
Borderline PT (03)		Benign PT = (01)			-		01			-		01		-		-		01			-		-	
		Borderline PT = (01)			-		01			-		01		-		-		-			01		-	
		Borderline PT/ Malignant PT = (01)			01		-			-		-		01		-		-			01		-	
Malignant PT (06)		Malignant PT (06)			01		05			-		02		04		-		-			02		04	
Histopathology (n= 17)	**Cytopathology (n= 17)**		**Cytologic features**																					
			Epithelial Fragments					Stromal: Epithelial ratio					Single scattered stromal cells						Mitosis					
			Pattern of epithelial fragments	Number				SP	S=E		EP		<10%		10-30%		>30%		0	+		++		+++
				<5 (Few)		>5 (Many)																		
Benign PT (08)	Benign PT = (06)		Folded	06		-		01	02		03		-		06		-		06	-		-		-
	Fibroadenoma = (02)		Mono-layered	-		02		-	-		02		02		-		-		02	-		-		-
Borderline PT (03)	Benign PT = (01)		Wavy	-		01		-	01		-		01		-		-		1	-		-		-
	Borderline PT = (01)		Folded	01		-		1	-		-		-		01		-		-	1		-		-
	Borderline PT/ Malignant PT = (01)		Wavy	01		-		1	-		-		-		-		01		-	-		01		-
Malignant PT (06)	Malignant PT (06)		Wavy	04		02		06	-		-		-		01		05		-	01		03		02

PT= Phyllodes Tumor, SP= Stromal Predominant, S=E = Stromal= Epithelial, EP= Epithelial Predominant

**Table 4:** Criteria for phyllodes classification according to WHO (12)

Criteria	Benign	Borderline	Malignant
Stromal cellularity and atypia	Minimal	Moderate	Marked
Stromal overgrowth	Minimal	Moderate	Marked
Mitosis/ 10 high power field	0-4	5-9	≥10
Tumor margins	Well-circumscribed with pushing tumor margins	Zone of microscopic invasion around tumor margins	Infiltrative tumor margins

## Conclusion

Cytological features of benign phyllodes tumor and fibroadenoma show significant overlap, whereas those of malignant phyllodes tumors are quite characteristic; however, it is challenging to diagnose and subclassify phyllodes tumors correctly on cytology. Moreover, rarity of phyllodes tumors makes the scenario even more difficult. In the present study, only a limited number of cases were studied, which found only the proportion of singly scattered stromal cells to be a statistically significant feature helpful in diagnosing and discriminating between different types of phyllodes tumors. However, there is a conspicuous lack of well-defined cytologic criteria to correctly differentiate the two fibroepithelial tumors that exhibit drastically different clinical behavior. Compared to core needle biopsy, fine needle aspiration (FNA) is a simpler, more affordable, and less invasive procedure. Therefore, further studies with larger sample sizes are needed to enhance the diagnostic accuracy of FNA in identifying phyllodes tumors. 
